# Microbial-Maximum Likelihood Estimation Tool for Microbial Quantification in Food From Left-Censored Data Using Maximum Likelihood Estimation for Microbial Risk Assessment

**DOI:** 10.3389/fmicb.2021.730733

**Published:** 2021-12-24

**Authors:** Gyung Jin Bahk, Hyo Jung Lee

**Affiliations:** ^1^Department of Food and Nutrition, Kunsan National University, Gunsan, South Korea; ^2^Department of Biology, Kunsan National University, Gunsan, South Korea

**Keywords:** microbial measurement, microbial censored data, non-detection (ND), limit of quantification (LOQ), Excel spreadsheet, microbial risk assessment (MRA)

## Abstract

In food microbial measurements, when most or very often bacterial counts are below to the limit of quantification (LOQ) or the limit of detection (LOD) in collected food samples, they are either ignored or a specified value is substituted. The consequence of this approach is that it may lead to the over or underestimation of quantitative results. A maximum likelihood estimation (MLE) or Bayesian models can be applied to deal with this kind of censored data. Recently, in food microbiology, an MLE that deals with censored results by fitting a parametric distribution has been introduced. However, the MLE approach has limited practical application in food microbiology as practical tools for implementing MLE statistical methods are limited. We therefore developed a user-friendly MLE tool (called “Microbial-MLE Tool”), which can be easily used without requiring complex mathematical knowledge of MLE but the tool is designated to adjust log-normal distributions to observed counts, and illustrated how this method may be implemented for food microbial censored data using an Excel spreadsheet. In addition, we used two case studies based on food microbial laboratory measurements to illustrate the use of the tool. We believe that the Microbial-MLE tool provides an accessible and comprehensible means for performing MLE in food microbiology and it will also be of help to improve the outcome of quantitative microbial risk assessment (MRA).

## Introduction

A large number of experiments on the microbiological status of various foods and food products are carried out globally. These experiments involve the collection of large amounts of data. However, in attempts to estimate the concentration of various microorganisms in food samples, those present in quantities below the detection limit are either ignored or a specified value is substituted. The statistical term for such results is “censored data,” i.e., non-zero values which cannot be measured, but are known to be below some threshold level (Hornung and Reed, 1990). Moreover, in food microbiology, since these low bacterial counts are compared to the limit of quantification (LOQ) or the limit of detection (LOD) of the method of analysis, and not reported if found to be lower than these values, only a limited amount of data is available in most cases (Busschaert et al., 2010). In food microbial measurements, there were found to contain some values below the LOQ or LOD of the sampling and analytical methods, and some were very heavily censored; over 90% of the data were below the LOQ in some enumeration data sets (i.e., quantitative methods), with nearly 100% (i.e., totally left-censored results) being lower than the LOD in presence/absence tests (i.e., qualitative methods). When quantification of the microorganisms in these samples is not possible, and assumed positive samples fall below the LOQ or LOD, they are either ignored or a specified value is substituted at or below the LOQ or LOD (Hewett and Ganser, 2007; Lorimer and Kiermeier, 2007). The consequence of these approaches is that they may lead to the over or underestimation of quantitative results. As an example, Lorimer and Kiermeier (2007) and Busschaert et al. (2010) showed that the difference in quantitative results depending on whether or not censored data are considered. It is necessary, therefore, to use a method for calculating the parameters characterizing the statistical distribution, for example, the arithmetic mean exposures that considers the food microbial censored data.

A maximum likelihood estimation (MLE) approach can be applied to deal with these kinds of censored data sets. Hornung and Reed (1990) and Helsel (2005) previously published and implemented an analysis of methods, in which the techniques proposed included an MLE statistical method for estimating dataset descriptors in the presence of non-detectable values in environmental hygiene and chemistry analyses. Recently, in food microbiology, an MLE method that deals with food microbial censored results by fitting a parametric distribution has been introduced for analyzing data with microbial censored observations (Shorten et al., 2006; Lorimer and Kiermeier, 2007; Busschaert et al., 2010, 2011; Chik et al., 2018). These researchers suggested this MLE method to deal with non-detected microbes in food microbiological test results, and focused primarily on applying MLE to deal with quantitative data that are censored on one side due to an LOQ or LOD (Busschaert et al., 2010; Wang and Gui, 2020). This MLE approach can contribute significantly to the quantification of microbial censored data. Furthermore, using censored data is becoming increasingly important as quantitative microbial risk assessment (MRA) methodologies continue to make greater use of quantitative data (Lorimer and Kiermeier, 2007).

However, the current maximum likelihood approach has limited practical application in food microbiology, or in the food industry, and there exists little practical support in terms of implementing the suggested MLE statistical methods. In addition, it is difficult to confirm the results of MLE actually used in food microbial prevalence studies. In order to address these limitations, Lorimer and Kiermeier (2007) suggested using an Excel program (which, at the time, would have been difficult to implement) or a statistical package, such as free and open source statistical software. Boysen et al. (2013) also reported implementation of an MLE approach for estimating the normal distribution parameters using the Solver add-in for Excel 2010. However, this approach was only used for MRA self-performance, and was not released as a publicly available tool. Therefore, there seems to be no dedicated tool that can be used to easily implement MLE-based methods in food microbiology. Considering the growing number of people and food industries using MLE on censored data, the need for user-friendly MLE tools has become increasingly important.

The objective of this study was to develop a user-friendly MLE tool, which could be easily used in food microbiology without the need for understanding the underlying mathematical concepts. Here we report the development the Microbial-MLE tool, using the Solver add-in for Excel 2016. In addition, we illustrated approaches using this tool in case studies based on food microbial laboratory measurements. We believe the Microbial-MLE tool provides an accessible and easily comprehensible means of performing MLE analyses of food microbial censored data.

## Materials and Methods

### Microbial-Maximum Likelihood Estimation Tool Configuration

**FIGURE 1:**
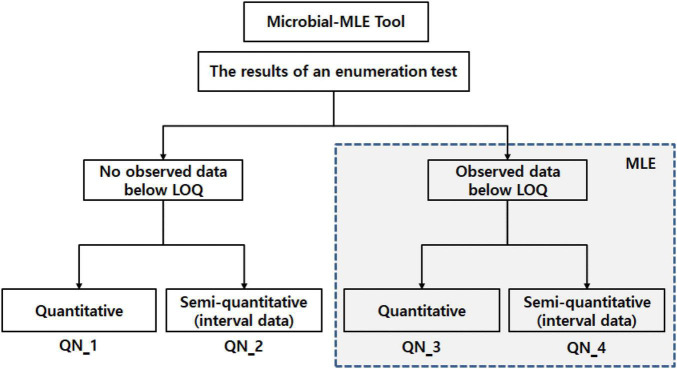
Configuration of the Microbial-MLE tool composed of four sub-tools (QN_1, 2, 3, and 4), the blue dashed line indicates the steps in which MLE is used. LOQ, limit of quantification; MLE, maximum likelihood estimation. (Details for QN_1 and 2 can be seen in the Excel program in the supplement, QN_3 and 4 are shown in Figures 3, 4).

The Microbial-MLE tool, included MLE techniques, was implemented in the Excel program with the Excel Solver add-in. As shown in Figure 1, the tool is composed of four sub-tools (QN_1, 2, 3, and 4), according to the type of microbiological enumeration test employed, i.e., whether data below the LOQ exists, and whether the data format is quantitative or semi-quantitative (interval data). QN_1 and 2 employ a general microbial concentration calculation method (i.e., no need to use MLE), and QN_3 and 4 are mainly used for MLE because of data occurring which is below the LOQ.

### Maximum Likelihood Estimation for Microbial Censored Data

Maximum likelihood estimation is a method for estimating the parameters (e.g., mean and standard deviation) of a statistical distribution from observed data (Finkelstein and Verma, 2001) and is also used to fit a statistical distribution to a set of food microbial censored data (Busschaert et al., 2010). The method of MLE, assuming an underlying normal distribution for the logarithm 10 concentration (i.e., lognormal distribution), may be used to estimate the means and standard deviations for microbial censored data (Lorimer and Kiermeier, 2007).

The lognormal distribution has two parameters, the mean (μ) and the standard deviation (σ). Let ln(*x*_*i*_) be the logarithm of the observed data value, *x*, of microbial sample *i*. Then, the probability distribution is defined by:


(1) $$N\,\left(x_{i},\mu ,\sigma \right)=\frac{1}{\sqrt{2\,\Pi }\,\sigma }\,\text{exp}\,\left[\frac{-{\left(\ln\left(x_{i}\right)-\mu \right)}^{2}}{2\,\sigma^{2}}\right]$$

If there are thus *n* observations, *y*_1_ = ln(*x*_1_), *y*_2_ = ln(*x*_2_) …and *y*_*n*_ = ln(*x*_*n*_), from a lognormal distribution with the mean (μ) and the standard deviation (σ), the probability (*P*_*N*_) of obtaining these values for the *n* observations is:


(2) $$P_{N}\,\left(x_{1},\ldots ,x_{n}|\mu ,\sigma \right)=\prod_{i=1}^{n}N\,\left(x_{i},\mu ,\sigma \right)$$

In the enumeration test result, if the LOQ or LOD is *DL* (CFU/g or ml) with non-detectable observations (*m*), the probability of observing a value less than *DL* is *P*_*DL*_, in a normal distribution with mean (μ) and standard deviation (σ).


(3) $$P_{D\,L}={\left(\int_{-\infty }^{D\,L}N\,\left(x;\mu ,\sigma \right)\,\text{ds}\right)}^{m}$$

The probability of the microbial population distribution parameters mean (μ) and standard deviation (σ), given the observed data (*n*) and non-detectable observations (*m*), is defined by Finkelstein and Verma (2001); Shorten et al. (2006), and Hewett and Ganser (2007):


(4) $$P\left(\mu ,\sigma |{\left\{x_{i}\right\}}_{i=1}^{n},m\right)=P_{D\,L}\times P_{N}$$

In statistical terminology this probability is called a likelihood, and the method of MLE finds those values of mean (μ) and the standard deviation (σ) that maximize this probability (Finkelstein and Verma, 2001).

### Maximum Likelihood Estimation in Excel for Microbial Censored Data

**FIGURE 2:**
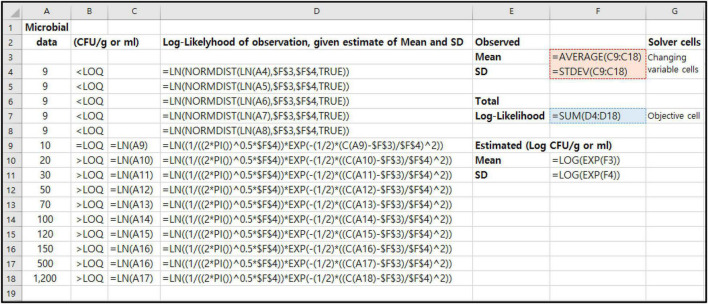
An Excel spreadsheet showing the structure of a template for Microbial MLE tool for an example of quantitative microbial censored data [*n* = 15, LOQ = 10 CFU/g or ml, Censored data% = 33.3%, (= 5/15)].

We show how the previous MLE-related formulas (Eqs 1–4) are represented in Excel (Microsoft Excel 2016; Microsoft Corp., Redmond, WA, United States) spreadsheet (Figure 2). These probabilities (*P*_*DL*_, *P*_*N*_, and *P*) are programmed into spreadsheet. In the case of *P*_*DL*_, in Excel, the function is NORMDIST. This function returns the normal cumulative distribution for the specified mean and standard deviation. Figure 2 shows the maximization of the likelihood function using microbial censored data (Column A). The values in F3 and F4 as changing variable cells in Solver tool are the mean and standard deviation of the logarithms (Column C9:C18) of the observed microbial data values in Column A. The Solver tool in Excel will select the proper values in E5 and E6 to maximize the sum of the log-Likelihoods, which can be found in cell F7 as objective cell in Solver tool. In this Microbial MLE Tool, all of these calculations and processes were automated with the Excel macro functions.

### Use of Microbial Maximum Likelihood Estimation Tool

**FIGURE 3:**
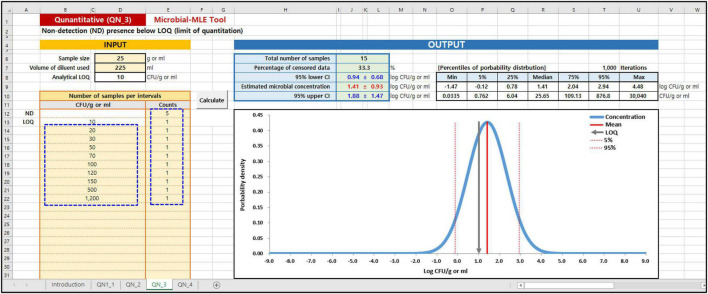
Input and Output panels of the Microbial-MLE tool for quantitative data (QN_3). Data is entered into the pale-yellow cells (blue dashed lines). The entry values surrounded by blue dashed lines are the hypothetical data.

**FIGURE 4:**
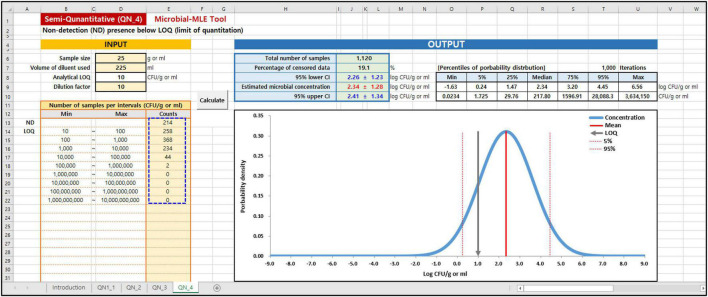
Input and Output panels of the Microbial-MLE tool for semi-quantitative (interval) data (QN_4). Data is entered into the pale-yellow cells (blue dashed line). The values shown are those used in case 1 (Table 1).

In the Microbial MLE tool, each of these sub-tools is implemented in one spreadsheet and is divided into an input and an output part. As shown in the blue dashed lines on the left in Figures 3, 4, the main input part consists of three input boxes: (1) sample size (g), (2) the volume of diluent used (ml), and (3) the observed measurement data, input as the results of quantitative (Figure 3) or semi-quantitative (Figure 4) enumeration tests (CFU/g or ml). Additionally, for QN_2 and 4 (interval data) there is an input for the dilution factor. Using (1) and (2) values, the LOQ is automatically calculated assuming a plating volume of 1 ml. After inputting these data, the “Calculate” button was clicked. The calculated results immediately appeared in the right-hand Output panel, as shown in Figures 3, 4. In the output panel, the following information is displayed: estimated microbial concentration as mean and standard deviation (SD) with 95% confidence intervals (CI) (Log CFU/g or ml), and a plot showing the probability distribution for the concentration, displaying the mean, LOQ value, 5th and 95th percentile. All of the above can be found in the Excel sheet (Supplementary Data: Microbial-MLE Tool.xlsm) attached to this article.

## Results

To show the result of Microbial-MLE tool, we used hypothetical data (presented in Figure 3) as an example for analyses involving quantitative left-censored data. This hypothetical data set comprises quantitative results with an LOQ of 10 CFU/g. In 5 of the 15 measurements (33%), the result is left-censored due to the LOQ. Using this tool, a normal distribution is estimated for these including censored data with 1.41 ± 0.93 log CFU/g as mean and standard deviation (Figure 3).

We illustrated, as case studies, microbial concentration estimation of left-censored food microbial data published in the literature (Jang et al., 2013; Chai et al., 2017), based on laboratory measurements using the developed Microbial-MLE tool. These data represented a variety of foods and microorganisms, and consisted mostly of semi-quantitative (interval) data. The LOQ of all these data sets was 10 CFU/g. The results of the estimated microbial concentrations using these data and the Microbial-MLE tool are shown in Table 1.

**TABLE 1:** The results of the estimated mean and standard deviation (SD) using the Microbial-MLE tool for four data sets (mainly semi-quantitative results) used as case studies.

Case number	Microorganisms	No. of samples (CFU/g)						Total	Reported Mean ± SD (log CFU/g)	Estimated Mean ± SD using this tool (log CFU/g)
		
		ND^1^	10∼10^2^	10^2^∼10^3^	10^3^∼10^4^	10^4^∼10^5^	10^5^∼10^6^			
Case 1	Total coliforms	214	258	368	234	44	2	1,120	–	2.34 ± 1.28
Case 2	*B. cereus*	1,008	49	48	12	2	1	1,120	–	−2.76 ± 2.93
Case 3	Total coliforms	16	19	34	22	8	1	100	2.23 ± 1.32	2.57 ± 1.34
Case 4	*E. coli*	81	16	2	1			100	0.37 ± 0.35	−0.44 ± 1.63

*^1^ND, not detected; LOQ, 10 CFU/g.*

In cases 1 and 2, the results of the total coliforms and *Bacillus cereus* analyses in sandwiches (Jang et al., 2013), which were produced on-site and served in bakeries, cafe’s, and sandwich bars in South Korea, were evaluated. The left-censored data due to the LOQ were in 214 (19%) and 1,008 (90%) of 1,120 samples, in the total coliforms and *B. cereus* analyses, respectively. Using this tool, the logarithms of the including censored data of total coliforms and *B. cereus* have been estimated to have a normal distribution with 2.34 ± 1.28 and −2.76 ± 2.93 log CFU/g, respectively. In particular, the result of *B. cereus* showed a high censored percentage (90%), and a large SD with a wide distribution of up to 10^5^∼10^6^ CFU/g due to the presence of outliers.

Cases 3 and 4 consist of 100 measurements of the total coliforms and *Escherichia coli* in retail beef samples (Chai et al., 2017). The left-censored data due to the LOQ were in 16 (16%) and 81 (81%) of 100 samples, in the total coliforms and *E. coli* analyses, respectively. Using this tool, the logarithmic values of the analysis results are fitted to a normal distribution with 2.57 ± 1.34 and −0.44 ± 1.63 log CFU/g, respectively.

**FIGURE 5:**
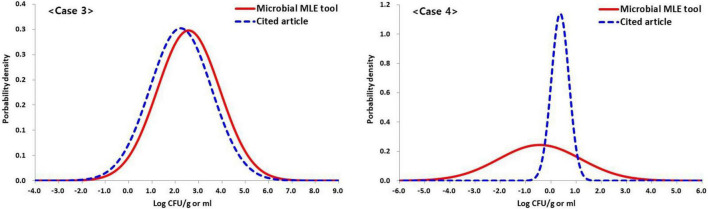
Based on case 3 and three data set (*n* = 100, LOQ = 10 CFU/g) in Table 1, comparison of the results of estimated concentration of the total coliforms (case 3) and *E. coli* (case 4) in retail beef samples using the Microbial-MLE tool and the results presented in the cited literature (Chai et al., 2017) (Case 3: 2.57 ± 1.34 and 2.23 ± 1.32 log CFU/g, Case 4: –0.44 ± 1.63 and 0.37 ± 0.35 log CFU/g, estimated by the Microbial-MLE tool and in the cited article, respectively, and percentage of censored data in Case 3 and 4 are 16 and 81%, respectively). The larger the censored rate, the greater the difference, i.e., these results show the difference in quantitative results depending on whether or not censored data are included.

Cases 1 and 2 did not show self-estimated quantitative results in the reference article and were not compared with the estimated values with this tool. However, in cases 3 and 4 were presented self-estimated quantitative results (Table 1). The estimated mean and SD values (2.57 ± 1.34 log CFU/g) changed very little due to the small amount of censored data, in the order of 2.23 ± 1.32 log CFU/g, in the results of the total coliforms, while the estimated mean and SD values (−0.44 ± 1.63 log CFU/g) changed greatly from 0.37 ± 0.35 log CFU/g due the abundance of censored data in the results of the *E. coli* analysis (Figure 5 and Table 1). This result shows the difference between the quantitative results when non-detected (left-censored) data is included and when it does not. Moreover, as shown in Figure 5 (case 4), the result (0.37 ± 0.35 log CFU/g) does not converge to the maximum value given in Table 1.

## Discussion

To increase the use of MLE in food microbial measurements, a user-friendly Microbial-MLE tool based on an MLE statistical method was developed. This tool estimates the quantitative concentration levels of microorganisms using food microbial censored data from the results of laboratory measurements. The tool works in an easy-to-use Excel spreadsheet and does not require complex mathematical knowledge about MLE on the part of the user. Thus, using this tool, anyone can rapidly and easily estimate the concentration of microorganisms from a variety of measurement results, or from routine monitoring of foodborne pathogens in various foods and food products.

Microbiological tests are generally divided into qualitative (presence/absence test) and quantitative (enumeration test) methods (Jarvis, 2008). Qualitative methods are concerned with investigating the presence or absence of a particular pathogen, such as specific foodborne pathogens (e.g., *Salmonella* spp. and *E. coli* O157:H7), even though quantitative method are available. On the other hand, quantitative methods are concerned with estimating microorganism concentrations, which may include total aerobic bacteria, coliforms and *E. coli*, as well as specific foodborne pathogens, such as *Staphylococcus aureus* and *B. cereus*. Currently, the tool we developed is only applicable to quantitative enumeration measurement results, and has not yet been applied to qualitative (presence/absence) test results possessing completely left-censored data (i.e., 100% censored data).

Microbial risk assessment is designed to quantitatively predict the probability of specific foodborne illness, such as pathogenic *E. coli* infections and salmonellosis, due to presence of causative pathogenic agents in the food products (Romero-Barrios et al., 2013). Thus, MRA have a requirement for quantitative data on the concentration of foodborne pathogens (Cassin et al., 1998; Boysen et al., 2013; Duarte et al., 2015), as microbiological contamination levels are often associated with predicted risk (Busschaert et al., 2010). The MLE method, which estimates values for the parameters that are most likely to have generated the observed measurements, can contribute to improving of the estimation for the concentration of microorganisms in foods, which is an important element of quantitative MRA.

The maximum likelihood method has been shown to produce unbiased estimates of both the mean and SD under a variety of conditions (Finkelstein and Verma, 2001). Moreover, the application of this MLE technique on microbial censored data has already been demonstrated to produce accurate and reliable results in food microbiology (Shorten et al., 2006; Lorimer and Kiermeier, 2007; Busschaert et al., 2010, 2011). However, despite many studies, the use of MLE method in food microbiology was, until recently, impractical, as MLE statistical methods are somewhat complex, and there was a lack of applicable tools with which to perform the necessary analyses. Previously, Lorimer and Kiermeier (2007) reported that MLE calculations could be performed by coding the methods manually and by using the Excel’s Solver add-in. In addition, the “fitdistrplus” R-package is available and allows also fitting statistical distributions to datasets containing censored data (Pouillot and Delignette-Muller, 2010). The estimation results of our Microbial-MLE tool and this R-package were exactly the same. Cases involving more complex data sets (e.g., 100% censored data) require more complex models, e.g., combinations of MLE and bootstrapping methods (Busschaert et al., 2010), and zero-inflated Poisson models (Gonzales-Barron et al., 2010; Duarte et al., 2015). Currently, however, MLE is easily implemented in commonly available spreadsheet software such as Excel. We demonstrate how this MLE method may be implemented using Excel spreadsheet. Once the spreadsheet template is set up, it can be readily used to estimate the concentrations of microorganisms from microbial censored data sets.

## Conclusion

In conclusion, our newly developed Microbial-MLE tool is simple to use and can rapidly estimate the best estimation concentrations of microorganisms from food microbial censored data, even if the user does not have deep knowledge of MLE. When reporting microbial measurement results, this tool can use censored data for analyzing the effectiveness of microbial interventions (Lorimer and Kiermeier, 2007). In addition, the tool will help improve the outcome of quantitative MRAs and can also be used as an educational tool for demonstrating MLE methods. However, this tool used only the parameters presented and did not take into account other parameters. Additionally, as mentioned above, the current tool is only available for quantitative enumeration test results and has not yet been applied to qualitative presence/absence test results, which are used for detecting the presence of most important foodborne pathogens. For use in various microbiological methods, future improvement and supplementation of to this tool should be undertaken.

## Data Availability Statement

The original contributions presented in the study are included in the article/Supplementary Material, further inquiries can be directed to the corresponding author.

## Author Contributions

GB and HL conceived of the study and participated in its design and coordination. Both authors reviewed and approved the final manuscript.

## Conflict of Interest

The authors declare that the research was conducted in the absence of any commercial or financial relationships that could be construed as a potential conflict of interest.

## Publisher’s Note

All claims expressed in this article are solely those of the authors and do not necessarily represent those of their affiliated organizations, or those of the publisher, the editors and the reviewers. Any product that may be evaluated in this article, or claim that may be made by its manufacturer, is not guaranteed or endorsed by the publisher.

## Abbreviations

CI: confidence intervals
LOD: limit of detection
LOQ: limit of quantification
MLE: maximum likelihood estimation
MRA: microbial risk assessment
ND: non-detection.
